# An Optical Sensor for Measuring the Position and Slanting Direction of Flat Surfaces

**DOI:** 10.3390/s16071061

**Published:** 2016-07-09

**Authors:** Yu-Ta Chen, Yen-Sheng Huang, Chien-Sheng Liu

**Affiliations:** 1 Department of Mechanical Engineering and Advanced Institute of Manufacturing with High-Tech Innovations, National Chung Cheng University, Chiayi County 62102, Taiwan; d04420006@ccu.edu.tw (Y.-T.C.); g01420122@ccu.edu.tw (Y.-S.H.); 2 Graduate Institute of Opto-Mechatronics, National Chung Cheng University, Chiayi County 62102, Taiwan

**Keywords:** flat surfaces, autofocus, autocollimator

## Abstract

Automated optical inspection is a very important technique. For this reason, this study proposes an optical non-contact slanting surface measuring system. The essential features of the measurement system are obtained through simulations using the optical design software Zemax. The actual propagation of laser beams within the measurement system is traced by using a homogeneous transformation matrix (HTM), the skew-ray tracing method, and a first-order Taylor series expansion. Additionally, a complete mathematical model that describes the variations in light spots on photoelectric sensors and the corresponding changes in the sample orientation and distance was established. Finally, a laboratory prototype system was constructed on an optical bench to verify experimentally the proposed system. This measurement system can simultaneously detect the slanting angles (x, z) in the x and z directions of the sample and the distance (y) between the biconvex lens and the flat sample surface.

## 1. Introduction

Among the measurement methods used in present day production lines, the non-contacting measurement technique is widely applied [1,2]. This technique is mainly divided into two types: image-based measurement and optical measurement. Image-based measurement focuses on image processing of captured images. The biggest limitations of this method are its long system response time and low accuracy. For these reasons, the technique has been gradually replaced in recent years by optical measurement. In the field of optical measurements, the positioning technique is of central importance [3,4]. For the laser positioning measurement technique, our research team has proposed a series of innovative system frameworks and combined them with optical diffusers to suppress the geometrical fluctuations of the laser beam and to achieve long-range and high-accuracy measurements [5,6,7]. Taken together, it is clear that optical measurement systems have the advantages of high measurement speed and accuracy.

In recent years, the positioning and slanting of a plane specular sample to be measured or processed has an important requirement in many fields of research and industry [8]. In applications of flat surface measurement techniques, Lee and Chang developed a laser scanning sensor with multiple position-sensitive device (PSD) detectors that can digitize a freeform surface through mathematical calculations using the Lambert model and geometric triangulation measurements [9]. The unique characteristic of this system is the use of multiple PSD detectors to resolve the dead zone of the measured angle and to broaden its range. Shiou developed a detector for distance and slanting angle measurements that can obtain the flat surface distance and slanting angle of the sample after the data are processed by triangulation operations [10]. Hirata and Haraguchi irradiated a sample with an annular laser beam and found that the shape of the laser beam changes with different slanting angles of the sample; by analyzing these changes, information on the slanting angles of the sample could be obtained. Finally, Gao developed a two-dimensional angle probe to measure the flatness of large silicon wafers [11]. Moreover, many methods are available to measure the parameters of slanting flat surfaces. However, to the best of our knowledge, these systems have complicated structures and high manufacturing costs. At present, there is no available measurement system with a simple architecture and straightforward assembly that can simultaneously measure the tilt angle and position information of a flat surface. Therefore, the aim of this study is to propose a fire-new measurement system with a simple architecture, straightforward assembly, and lower manufacturing cost that can simultaneously detect the tilt angle and position information of the sample.

## 2. Architecture and Measurement Method of Proposed Measurement System

**Figure 1:**
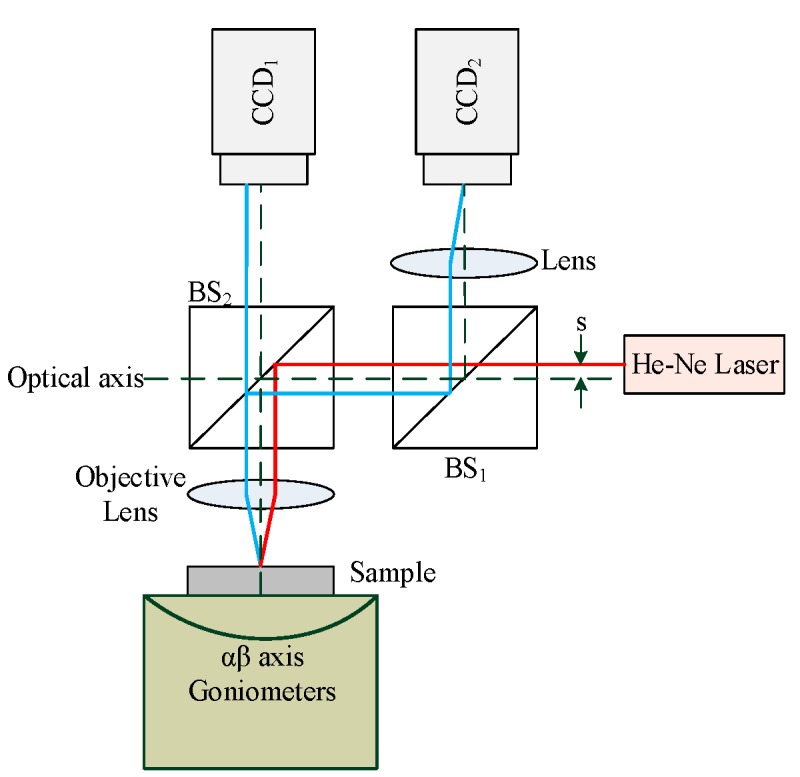
Architecture of proposed measurement system.

The architecture of the proposed measurement system in this study consists of a He-Ne laser as the light source, two beam splitters (BS) accompanied by two biconvex lenses, and two charge-coupled devices (CCD) that are used as the photoelectric sensors to receive the optical signals. As shown in Figure 1, this design is an adaptation of an auto-focusing technique, namely, the triangulation method. The laser beam is directed off the optical axis by a small distance (s) and is then reflected by BS_2_ to become incident on the sample through the biconvex lens. The laser beam is then reflected by the sample to pass through the biconvex lens back to BS_2_ where the beam was split into two beams. One of the split beams goes directly through BS_2_ and is incident on CCD_2_. Notably, the laser spots on CCD_2_ will change as a function of the different sample orientations and distance. The other beam is reflected by beam splitter BS_1_ and travels through the biconvex lens before proceeding to CCD_1_. The information of the laser spots on CCD_1_ will depend not only on the sample surface orientation but also on the change in the distance between the biconvex lens and the sample.

When using this proposed measurement system to measure the distance (*δ_y_*) between the biconvex lens the flat sample surface and the slanting angles in the *x* and *z* directions (*ω_x_*, *ω_z_*), the shape and center position of the laser beam received by the two CCDs will change as a function of both changes in the distance (*δ_y_*) and slanting angles (*ω_x_*, *ω_z_*). In addition, the received photoelectric signal contains factors that are influenced by the slanting angles (*ω_x_*, *ω_z_*) of the sample when the sample distance (*δ_y_*) resolved by the two CCDs is measured. Therefore, light beam tracing equations must be established via a HTM and skew-ray tracing to discuss the relationship between the sample distance (*δ_y_*), slanting angles (*ω_x_*, *ω_z_*), and the corresponding change in the center position of the laser spot. In this regard, a detailed mathematical derivation will be presented in Section 4.

## 3. Optical Simulation of Proposed Measurement System

The ray trace function of the commercially available optical simulation software Zemax (Radiant Zemax LLC, Redmond, WA, USA) was used to simulate the actual ray propagation in the proposed measurement system. The Matlab software (The MathWorks, Inc., Natick, MA, USA) was then used to process the images of the laser spot position map, to calculate the location of the spot center, and to plot graphs for the spot position versus sample surface orientation and distance. In doing so, the feasibility of the proposed measurement system is determined and the measurement trend is determined.

**Figure 2:**
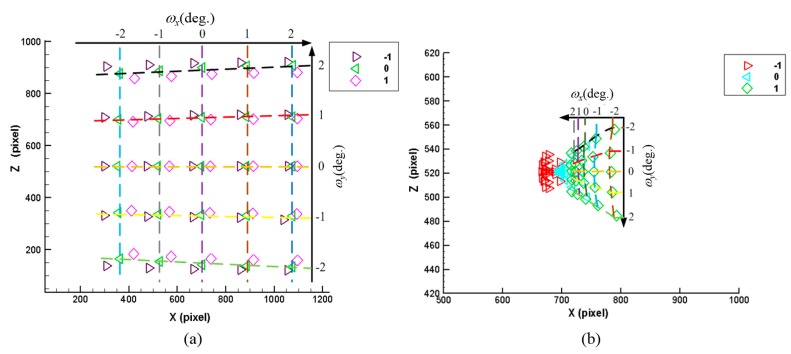
Optical simulation results: (**a**) changes in laser spots on CCD_1_ and (**b**) changes in laser spots on CCD_2_.

Figure 2 shows the changes in the laser spots on CCD_1_ and CCD_2_ when the sample distance (δy) is within the range of −1 mm~+1 mm and the slanting angles (*ω_x_*, *ω_z_*) are within the range of −2°~+2°. The figure indicates that the laser spots on CCD_1_ and CCD_2_ show a changing trend when the sample experiences a small change in angle and distance. However, the relationship is not linear, and thus, the sample distance and orientation cannot be determined directly from the simulation results. Therefore, it is necessary to derive a mathematical measurement equation to analyze the relation between the sample distance/orientation and the two CCDs to obtain a quantitative mathematical analysis of the proposed measurement system.

## 4. Mathematical Modeling of Proposed Measurement System and Derivation of Actual Ray Path

Mathematical modeling is established by using a HTM and the skew-ray tracing method proposed by Lin to model and perform ray tracing for the proposed measurement system architecture in this paper [12,13]. The HTM corresponding to the coordinate frame of each optical boundary relative to a reference coordinate system (*xyz*)_0_ is defined sequentially. A mathematical model is proposed to develop a systematic forward and reverse mathematical derivation. Then, a linear equation of the measurement system is obtained by using a first-order Taylor series expansion to evaluate the first-order optical properties of the proposed measurement system.

### 4.1. Establishing Optical Boundaries for Proposed Measurement System

**Figure 3:**
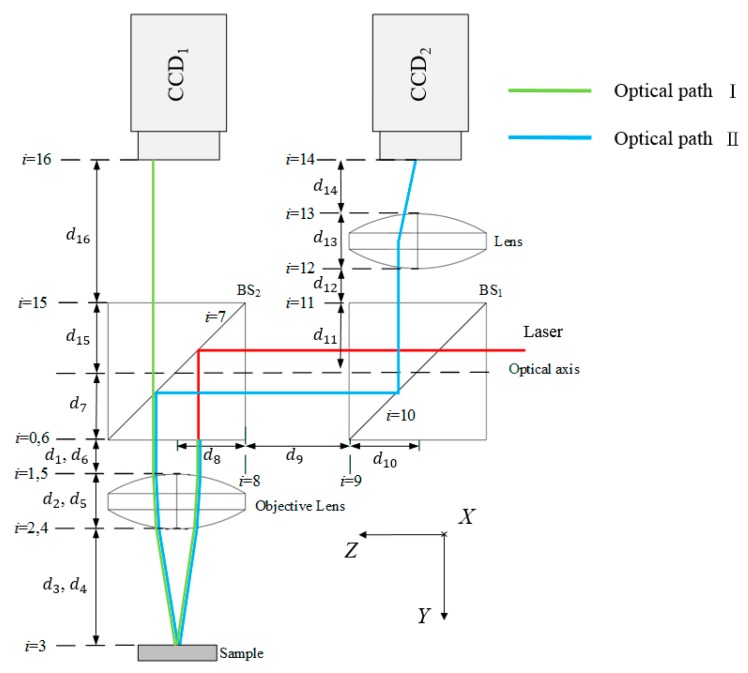
Definition of each optical boundary of the proposed measurement system.

Based on the modeling steps in the skew-ray tracing method proposed by Lin [12,13], the optical boundaries of the system ri¯i are first defined. The proposed measurement system contains two parts: ray path Ι, which contains a total of 14 optical boundaries, and ray path ΙΙ, which contains a total of eight optical boundaries, as shown in Figure 3:

(1) Ai¯0=[IixJixKixtixIiyJiyKiytiyIizJizKiztiz0001]

The transformation matrices of coordinate frames (*xyz*)*_i_* of each optical boundary ri¯i relative to the reference coordinate frame (*xyz*)_0_ are established as Equation (1) using the HTM. The elements in the HTM for each optical boundary are shown in Table 1 and Table 2. The notations C and S denote cosine and sine, respectively. As seen, given are the HTM tables of coordinate (*xyz*)*_i_* for each optical boundary ri¯i relative to the reference coordinate system of ray paths Ι and ΙΙ, respectively, where *ω_x_*, *ω_z_*, and *δ_y_* represent the rotation angle in the *x* and *z* directions and the distance (*δ_y_*) between the biconvex lens and the flat sample surface. The initial position of the laser light source is defined as *P_0_* = [0 0 − 2 1]^T^, and its unit vector is defined as $\ell_{0}$ = [0 1 0 0]^T^.

### 4.2. Incident Point and Unit Vector of Incident Laser Ray

**Figure 4:**
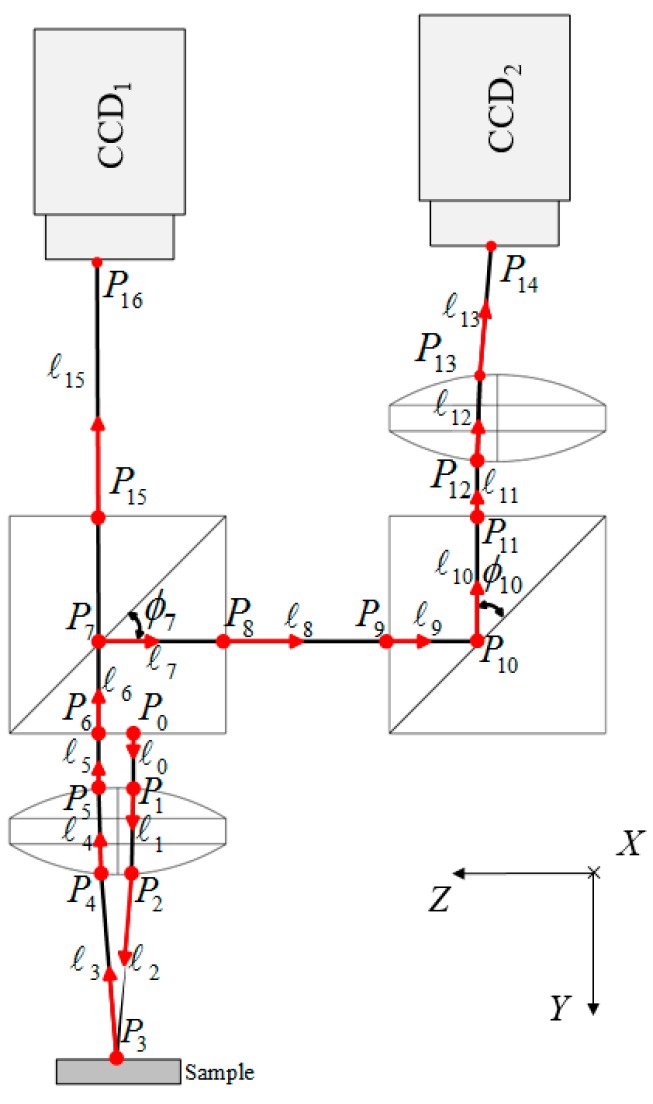
Definition of incident point and unit vectors of reflected and refracted rays.

To trace the propagation of the laser ray in the proposed measurement system, the initial position of the laser light source is set as $P_{0}$ = [0 0–2 1]^T^ and its unit vector is set as $\ell_{0}$ = [0 1 0 0]^T^. The laser ray begins at an initial position that is along the unit vector but incident to the proposed measurement system. For the actual ray propagation in the proposed measurement system, the ray incident point on each optical boundary, the unit vectors of the reflected rays [14], and the unit vectors of the refracted rays are shown in Figure 4 and can be obtained from Equations (2) to (8). Equation (2) gives the expressions of the optical boundary ri¯i and its unit normal vector ni¯i with parameters α_i_ and β_i_. Note that the non-negative parameter *λ_i_* represents the geometrical length from point $P_{i-1}$ to $P_{i}$. The ± sign in Equation (3) indicates there may be two possible intersection points of this ray and a complete sphere. Clearly, only one of these points is useful, thus the appropriate sign must be chosen. The spherical coordinates, α_i_ and β_i_, at the incidence point $P_{i}$ can be determined from Equation (6). Equations (7) and (8) define the *D_i_* and *E_i_* as the parameters in spherical and plane surface boundary situation, respectively.

The results of these calculations are described in Table 3 and Table 4. Table 3 and Table 4 are the calculated parameters of the ray incident points and ray unit vectors of ray path I and II, respectively.

*Incident point*

(2) $${\bar{P}}_{i}=\left[\begin{array}{c} P_{ix}\\ P_{iy}\\ P_{iz}\\ 1 \end{array}\right]=\left[\begin{array}{c} P_{i-1x}+\ell_{i-1x}\lambda_{i}\\ P_{i-1y}+\ell_{i-1y}\lambda_{i}\\ P_{i-1z}+\ell_{i-1z}\lambda_{i}\\ 1 \end{array}\right]=\left[\begin{array}{c} \sigma_{i}\\ \rho_{i}\\ \tau_{i}\\ 1 \end{array}\right]=\left[\begin{array}{c} \left|R_{i}\right|C\beta_{i}C\alpha_{i}\\ \left|R_{i}\right|C\beta_{i}S\alpha_{i}\\ \left|R_{i}\right|S\beta_{i}\\ 1 \end{array}\right]$$

(3) $$\lambda_{i}=-D_{i}\pm \sqrt{{D_{i}}^{2}-E_{i}}$$

Boundary equations

Spherical surface boundary:

(4) $$\begin{array}{c} D_{i}=\rho_{i}(-\ell_{i-1y})+\tau_{i}\ell_{i-1z}+P_{i-1x}\ell_{i-1x}+P_{i-1y}\ell_{i-1y}+P_{i-1z}\ell_{i-1z}\\ E_{i}=P_{i-1x}^{2}+P_{i-1y}^{2}+P_{i-1z}^{2}+\rho_{i}^{2}+\tau_{i}^{2}-{R_{i}}^{2}-2\left(\rho_{i}P_{i-1y}+\tau_{i}P_{i-1z}\right) \end{array}$$

Plane surface boundary:

(5) $$\begin{array}{c} D_{i}=n_{ix}P_{i-1x}+n_{iy}P_{i-1y}+n_{iz}P_{i-1z}+e_{i}\\ E_{i}=n_{ix}\ell_{i-1x}+n_{iy}\ell_{i-1y}+n_{iz}\ell_{i-1z} \end{array}$$

For the purpose of tracing the reflected or refracted ray incidents at the boundary surface, one needs the incidence angle *θ_i_* (0° ≤ *θ_i_* ≤ 90°), which is determined by the dot product of ${\bar{\ell }}_{i-1}$ and the active unit normal vector $\bar{n}$_i_ . Then the ray unit directional vector ${\bar{\ell }}_{i}$ can be obtained by the refraction and reflection law of optics, and Equations (7) and (8) show the calculated and simplified result of them. Note that *N_i_* is the index of refraction or reflection defined by the Snell’s law. More details about the skew-ray tracing method derivation process are given in publications by Lin [14,15].

(6) $$\begin{array}{l} C\theta_{i}=\left|{\bar{\ell }}_{i-1}•{\bar{n}}_{i}\right|=-{\bar{\ell }}_{i-1}•{\bar{n}}_{i}\\ \alpha_{i}=a\tan2(P_{i-1y}+\ell_{i-1y}\lambda_{i}+\rho_{i},\;P_{i-1x}+\ell_{i-1x}\lambda_{i})\\ \beta_{i}=a\tan2\left(P_{i-1z}+\ell_{i-1z}\lambda_{i}+\tau_{i},\;\sqrt{{\left(P_{i-1x}+\ell_{i-1x}\lambda_{i}\right)}^{2}+{\left(P_{i-1y}+\ell_{i-1y}\lambda_{i}+\rho_{i}\right)}^{2}}\right) \end{array}$$

*The unit vector of the reflected ray*:

(7) $${\bar{\ell }}_{i}=\left[\begin{array}{c} \ell_{ix}\\ \ell_{iy}\\ \ell_{iz}\\ 0 \end{array}\right]=\left[\begin{array}{c} \ell_{i-1x}+2C\theta_{i}n_{ix}\\ \ell_{i-1y}+2C\theta_{i}n_{iy}\\ \ell_{i-1z}+2C\theta_{i}n_{iz}\\ 0 \end{array}\right]={\bar{\ell }}_{i-1}+2C\theta_{i}n_{i}$$

*The unit vector of the refracted ray*:

(8) $${\bar{\ell }}_{i}=\left[\begin{array}{c} \ell_{ix}\\ \ell_{iy}\\ \ell_{iz}\\ 0 \end{array}\right]=\left[\begin{array}{c} -n_{ix}\sqrt{1-N_{i}^{2}+{\left(N_{i}C\theta_{i}\right)}^{2}}+N_{i}\left(\ell_{i-1x}+n_{ix}C\theta_{i}\right)\\ -n_{iy}\sqrt{1-N_{i}^{2}+{\left(N_{i}C\theta_{i}\right)}^{2}}+N_{i}\left(\ell_{i-1y}+n_{iy}C\theta_{i}\right)\\ -n_{iz}\sqrt{1-N_{i}^{2}+{\left(N_{i}C\theta_{i}\right)}^{2}}+N_{i}\left(\ell_{i-1z}+n_{iz}C\theta_{i}\right)\\ 0 \end{array}\right]=\left(N_{i}C\theta_{i}-\sqrt{1-N_{i}^{2}+{\left(N_{i}C\theta_{i}\right)}^{2}}\right)\bar{n_{i}}+N_{i}{\bar{\ell }}_{i-1}$$

Sequentially tracing the ray incident points on each optical boundary gives the coordinates of the spots on the two CCDs. Because *ω_x_*, *ω_z_* and *δ_y_* are generally very small, a first-order Taylor series expanded about *ω_x_* = *ω_z_* = *δ_y_* = 0 can be used to obtain a first-order equation of the proposed system measurement. Then, substituting into the equation the parameters for the actual sensor position and measurement system, such as the system internal unit distance *d_i_* and the lens surface radius *R_i_*, the system measurement Equation (9) of the spot coordinates on the two CCDs can be obtained:

(9) $$\left\{ \begin{array}{l} P_{14x}=215.147(3.44801-0.155909\delta_{y}+0.780777\omega_{x})\\ P_{14z}=214.876(2.69362+0.0402449\omega_{z})\\ P_{16x}=215.147(3.47192+0.464506\delta_{y}+48.5221\omega_{x})\\ P_{16z}=214.876(2.43596-50.5223\omega_{z}) \end{array}\right.$$

Equation (10) is the result of a reverse derivation of solving Equation (9). Equation (10) gives a set of linear equations that gives accurate sample slanting angles (*ω_x_*, *ω_z_*) and distance (*δ_y_*) from the spot center locations on the CCDs:

(10) $$\begin{array}{l} \omega_{x}=-0.27030+0.00027P_{14x}+0.00009P_{16x}\\ \omega_{z}=0.04822-\;0.00009P_{16z}\\ \delta_{y}=20.76186-0.02845P_{14x}+0.00046P_{16x} \end{array}$$

## 5. Experimental Results and Discussion

**Figure 5:**
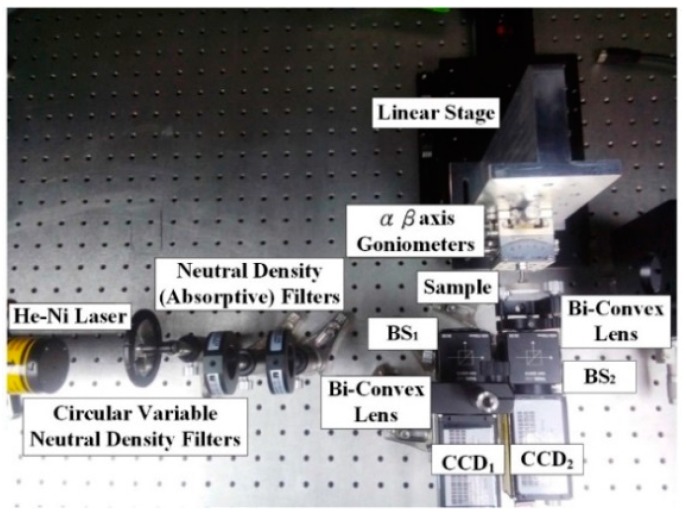
Photograph of the laboratory-built prototype.

Figure 5 shows the actual design of the proposed measurement system on an optical bench.

The experimental set-up includes placing the sample on a dual (α-β) axis goniometer that rotates about the α and β axes with a resolution of 0.1°. LabVIEW (National Instruments Co., Mopac Expwy Austin, TX, USA ) is used to control the linear stage (one step of 1 lm, HF-KP053-B series, Chiuan Yan Tech, Changhua, Taiwan) to induce mono-dimensional movements of the sample (i.e., distance (*δ_y_*)), and a He-Ne Laser is used as the system light source (*λ* = 632 nm, 2 mW).

### 5.1. Part 1

**Figure 6:**
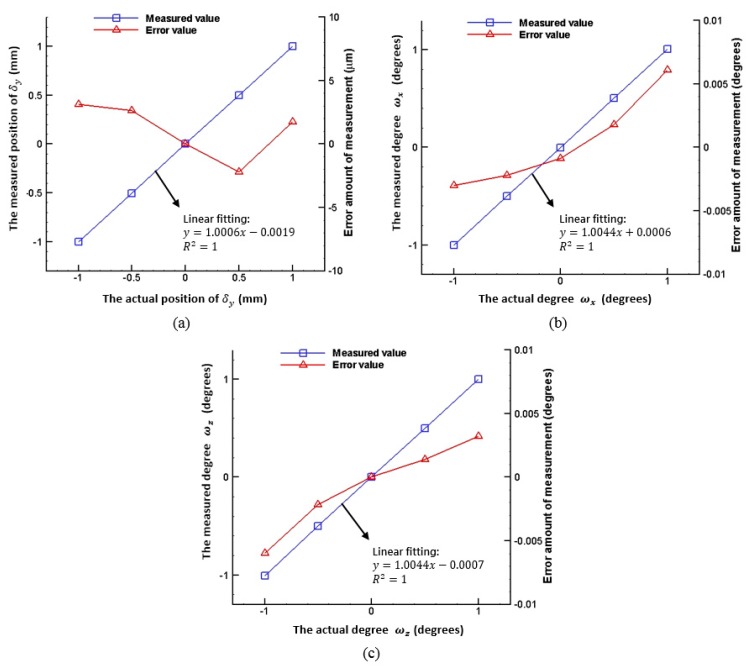
Verification of experimental results: (**a**) distance (*δ_y_*); (**b**) slanting angle (*ω_x_*); and (**c**) slanting angle (*ω_z_*).

The measurement results of the sample distance (*δ_y_*) and slanting angles (*ω_x_*, *ω_z_*) are verified individually. When verifying the measurement results of the distance (*δ_y_*), the sample is driven by a linear stage along the y-direction with both slanting angles (*ω_x_*, *ω_z_*) set to 0°. The measurement range of the distance is −1~+1 mm. When verifying the measurement results of the flat sample slanting angle *ω_x_* using a dual axis goniometer, the sample distance (*δ_y_*) is fixed to 0 mm, and the slanting angle *ω_z_* is set to 0°. The measurement range of the slanting angle is −1°~+1°. The verification method of the measurement results of the flat sample slanting angle *ω_z_* is similar to that of *ω_x_*. The verification results are shown in Figure 6. All measurement points in Figure 6 originate from simultaneous images captured by two CCDs. The sampling frequency is one image per second, and 20 images are captured per measured point. Then, the average values of the sample distance (*δ_y_*) and slanting angles (*ω_x_*, *ω_z_*) are calculated using the results of the mathematical derivations in the previous section. It is observed that the measured errors of the slanting angles (*ω_x_*, *ω_z_*) and the distance (*δ_y_*) are 0.009° and 6 µm, respectively. In other words, the measured linearity of the slanting angles (*ω_x_*, *ω_z_*) and the distance (*δ_y_*) is 0.45% and 0.3%, respectively.

### 5.2. Part 2

This part would show the complex measurement results of the sample distance (*δ_y_*) and slanting angles (*ω_x_*, *ω_z_*). However the possible permutations of the sample distance (*δ_y_*) and slanting angles (*ω_x_*, *ω_z_*) multiply towards infinity. This part would verify the measurement results of the distance (*δ_y_*) under the setting of slanting angles (*ω_x_*, *ω_z_*) that are equal to (2°, 2°), (−2°, −2°), (−2°, 2°), (2°, −2°). The purpose of this experiment is to find the limit of the laboratory-built prototype.

**Figure 7:**
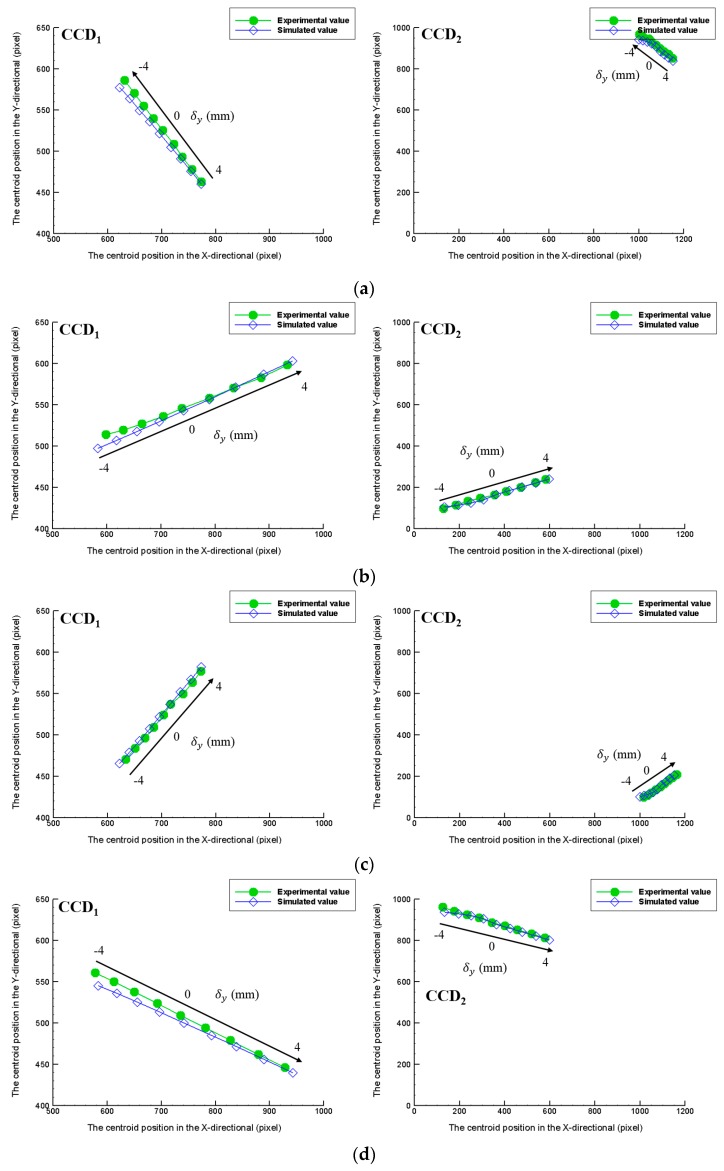
Verification of the distance (*δ_y_*) results with the slanting angles (*ω_x_*, *ω_z_*): (**a**) (2°, 2°), (**b**) (−2°, −2°), (**c**) (2°, −2°), and (**d**) (−2°, 2°).

The maximum measurement range of the distance (*δ_y_*) is −4~+4 mm and each measured point is 1 mm apart. The sampling frequency is one image per second, and 20 images are captured per measured point. All measurement points in Figure 7 originate from simultaneous images captured by two CCDs. Figure 7 shows the experimental results are very similar to the simulation results on two CCDs.

## 6. System Stability Test

**Figure 8:**
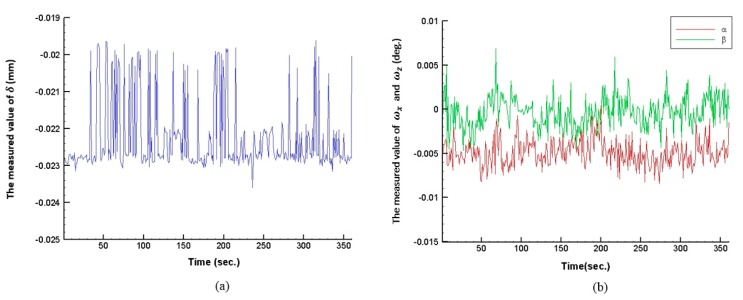
Results of proposed measurement system stability test: (**a**) distance (*δ_y_*) and (**b**) slanting angles (*ω_x_*, *ω_z_*).

The stability test involves first placing the proposed measurement system in a stable environment for at least 15 min to regulate the temperature of the system. Then, the sample is adjusted to a fixed orientation and distance (e.g., distance *δ_y_* = 0 mm; slanting angles *ω_x_* = *ω_z_* = 0°), and the system is sampled continuously for 6 min. The results of the system stability test are shown in Figure 8. As seen, system stability is achieved within 6 min at a distance and inline angle of 3 *μ*m and 0.01°, respectively. However the measured accuracy of the proposed measurement system is not only influenced by the laser beam instability but also by misalignments, aberrations, CCD sensitivity, etc. and can be enhanced by using high quality laser and close-loop with a feedback signal methods [16,17]. As a result, in the future, these factors will need to be considered and optimized to reduce system errors and improve measured accuracy. Also we will produce modified physics model for real applications in industry and we must overcome some additional challenges. In our plan, we will combine our previous study to improve robustness toward geometrical fluctuations of the laser beam of the proposed measurement system and analyze accuracy influences by the components position error, misalignments, aberrations, quantization errors, vibrations, and laser non-stability effect, etc. 

## 7. Comparison of Proposed Measurement System and Automatic Collimator

**Figure 9:**
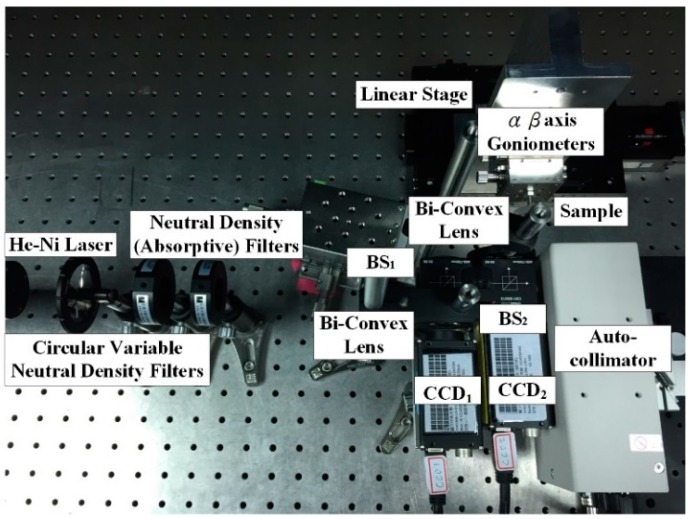
Set-up of the proposed measurement system and automatic collimator comparison test.

**Figure 10:**
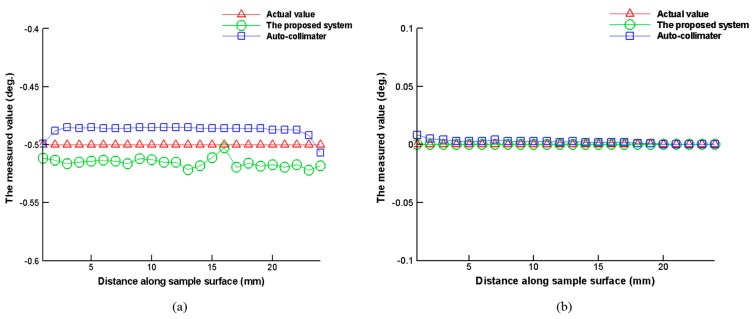
Comparative test results of proposed measurement system and automatic collimator: (**a**) *ω_x_* experimental results and (**b**) *ω_z_* experimental results.

We performed a comparison test using a commercially available automatic collimator (H400-C050, measurement range: ±0.5°, Suruga Seiki Co., Shizuoka, Japan) and the proposed measurement system to measure the slanting angles of the sample (*ω_x_*, *ω_z_*). As shown in Figure 9, the sample is placed on a dual axis goniometer that is installed on a linear stage. The proposed measurement system and the commercially available automatic collimator are configured alongside each other to monitor the slanting angles (*ω_x_*, *ω_z_*) of the sample on the linear stage simultaneously. The linear stage is driven so that the automatic collimator and the proposed measurement system can simultaneously measure the sample slanting angle information in scanning mode. Figure 10 shows the results of comparing the proposed measurement system and the conventional automatic collimator. Using the dual axis goniometer to set the sample slanting angles as *ω_x_* = 0.5° and *ω_z_* = 0°, one measurement is recorded for every 1 mm movement of the linear stage. Each recorded point is the averaged result of 10 separate measurements. The experimental results have shown that the proposed measurement system has excellent performance and its practicability, compared to the conventional automatic collimator.

## 8. Conclusions

An optical non-contacting measurement system has been successfully developed in this study. This system can measure workpiece samples with a slanting angle and simultaneously measure the sample distance and its orientation. The optical simulation software Zemax has been used to perform a feasibility analysis of this system. Additionally, a model of the proposed measurement system has been established using a HTM. The skew-ray tracing method is employed to trace the actual ray propagation path to obtain the system measurement equations. Finally, a laboratory-built prototype is constructed on an optical bench to verify the performance of the proposed measurement system. The orientation and distance measurement experiments show that the measuring ranges for the angles and distance are ±1° and ±1 mm, respectively, and the measured errors of the angles and the distance are 0.009° and 6 µm, respectively. In other words, the measured linearity of the slanting angles (*ω_x_*, *ω_z_*) and the distance (*δ_y_*) is 0.45% and 0.3%, respectively.

## Figures and Tables

**Table 1:** Parameters of the optical boundary coordinate transformation matrix for ray path I.

**I**	**I = 1**	**I = 2**	**I = 3**	**I = 4**	**I = 5**	**I = 6**	**I = 7**
**Type**	**Convex Spherical**	**Concave Spherical**	**Flat Mirror (Sample)**	**Convex Spherical**	**Concave Spherical**	**Flat Refracting**	**Flat Mirror**
N_i_	1/1.515	1.515	Reflected	1/1.515	1.515	1/1.515	
R_i_	24.397	24.397		24.397	24.397		
*I_ix_*	1	1	1	1	1	1	−1
*I_iy_*	0	0	S*ω_z_*	0	0	0	0
*I_iz_*	0	0	0	0	0	0	0
*J_ix_*	0	0	−C*ω_x_*S*ω_z_*	0	0	0	0
*J_iy_*	1	1	1	1	1	1	$-\sqrt{2}/2$
*J_iz_*	0	0	C*ω_x_*S*ω_z_*	0	0	0	$\sqrt{2}/2$
*K_ix_*	0	0	S*ω_x_*S*ω_z_*	0	0	0	0
*K_iy_*	0	0	S*ω_x_*C*ω_z_*	0	0	0	$\sqrt{2}/2$
*K_iz_*	1	1	1	1	1	1	$\sqrt{2}/2$
*t_ix_*	0	0	0	0	0	0	0
*t_iy_*	30.747	−7.915	38.202 + *δ_y_*	−7.915	30.747	0	−12.7
*t_iz_*	0	0	0	0	0	0	0
**I**	**I = 8**	**I = 9**	**I = 10**	**I = 11**	**I = 12**	**I = 13**	**I = 14**
**Type**	**Flat Refracting**	**Flat Refracting**	**Flat Mirror**	**Flat Refracting**	**Convex Spherical**	**Concave Spherical**	**CCD_1_**
N_i_	1.515	1/1.515	Reflected	1.515	1.515	1/1.515	
R_i_	0	0	0	0	24.397	24.397	0
*I_ix_*	−1	−1	−1	−1	−1	−1	−1
*I_iy_*	0	0	0	0	0	0	0
*I_iz_*	0	0	0	0	0	0	0
*J_ix_*	0	0	0	0	0	0	0
*J_iy_*	0	0	$\sqrt{2}/2$	−1	−1	−1	−1
*J_iz_*	−1	−1	$-\sqrt{2}/2$	0	0	0	0
*K_ix_*	0	0	0	0	0	0	0
*K_iy_*	−1	−1	$-\sqrt{2}/2$	0	0	0	0
*K_iz_*	0	0	$-\sqrt{2}/2$	1	1	1	1
*t_ix_*	0	0	0	0	0	0	0
*t_iy_*	−12.7	−12.7	−12.7	−25.4	−56.147	−17.485	−63.602
*t_iz_*	−12.7	−31.9	−38.1	−44.6	−44.6	−44.6	−44.6

**Table 2:** Parameters of the optical boundary coordinate transformation matrix for ray path II.

I	I = 1	I = 2	I = 3	I = 4	I = 5	I = 6	I = 15	I = 16
Type	Convex Spherical	Concave Spherical	Flat Mirror (Sample)	Convex Spherical	Concave Spherical	Flat Refracting	Flat Refracting	CCD_2_
N_i_	1/1.515	1.515	Reflected	1/1.515	1.515	1/1.515	1.515	
R_i_	24.397	24.397		24.397	24.397			
*I_ix_*	1	1	1	1	1	1	−1	−1
*I_iy_*	0	0	S*ω_z_*	0	0	0	0	0
*I_iz_*	0	0	0	0	0	0	0	0
*J_ix_*	0	0	−C*ω_x_*S*ω_z_*	0	0	0	0	0
*J_iy_*	1	1	1	1	1	1	−1	−1
*J_iz_*	0	0	C*ω_x_*S*ω_z_*	0	0	0	0	0
*K_ix_*	0	0	S*ω_x_*S*ω_z_*	0	0	0	0	0
*K_iy_*	0	0	S*ω_x_*C*ω_z_*	0	0	0	0	0
*K_iz_*	1	1	1	1	1	1	1	1
*t_ix_*	0	0	0	0	0	0	0	0
*t_iy_*	30.747	−7.915	38.202	−7.915	30.747	0	−25.4	−49.426
*t_iz_*	0	0	0	0	0	0	0	0

**Table 3:** Ray incident points and ray unit vector parameters of ray path I.

**I**	**I = 1**	**I = 2**	**I = 3**	**I = 4**	**I = 5**	**I = 6**	**I = 7**
**Type**	**Convex Spherical**	**Concave Spherical**	**Flat Mirror (Sample)**	**Convex Spherical**	**Concave Spherical**	**Flat Refracting**	**Flat Refracting**
N_i_	1/1.515	1.515	Reflected	1/1.515	1.515	1/1.515	Reflected
$\phi_{i}$	0	0	0	0	0	0	π/4
*n_ix_*	Cβ_1_Cα_1_	Cβ_1_Cα_1_	−C*ω_x_*S*ω_z_*	Cβ_4_Cα_4_	Cβ_5_Cα_5_	0	0
*n_iy_*	Cβ_1_Cα_1_	Cβ_1_Cα_1_	C*ω_x_*C*ω_z_*	Cβ_4_Cα_4_	Cβ_5_Cα_5_	1	$S\phi_{7}$
*n_iz_*	Sβ_1_	Sβ_1_	S*ω_x_*	Sβ_4_	Sβ_5_	0	$-C\phi_{7}$
*ρ_i_*	−(d_1_ + R)	R − (d_1_ + d_2_)	0	R − (d_1_ + d_2_)	−(d_1_ + R)	0	0
*τ_i_*	0	0	0	0	0	0	0
*e_i_*	Sphere	Sphere	−(d_1_ + d_2_ + d_3_)	Sphere	Sphere	0	$-d_{7}S\phi_{7}$
**I**	**I = 8**	**I = 9**	**I = 10**	**I = 11**	**I = 12**	**I = 13**	**I = 14**
**Type**	**Flat Refracting**	**Flat Refracting**	**Flat Mirror**	**Flat Mirror (Sample)**	**Convex Spherical**	**Concave Spherical**	**CCD_1_**
N_i_	1.515	1.515	Reflected	1.515	1/1.515	1.515	1/1.515
$\phi_{i}$	0	0	3π/4	0	0	0	
*n_ix_*	0	0	0	0	Cβ_5_Cα_5_	0	1
*n_iy_*	0	0	$C\phi_{10}$	1	Cβ_5_Cα_5_	1	0
*n_iz_*	1	1	$S\phi_{10}$	0	Sβ_5_	0	0
*ρ_i_*	0	0	0	0	$d_{7}S2\phi_{10}-d_{8}C2\phi_{10}$$+d_{11}+d_{12}+R$	d_7_ + d_11_ + d_12_ + d_13_ − R	0
*τ_i_*	0	1	0	0	$d_{7}C2\phi_{10}+d_{8}S2\phi_{10}$$+d_{10}+d_{9}$	d_8_ + d_9_ + d_10_	1
*e_i_*	−d_8_	−(d_8_ + d_9_)	$(d_{8}+d_{9}+d_{10})S\phi_{10}$$-d_{7}C\phi_{10}$	$d_{8}C2\phi_{10}-d_{11}$$-d_{7}S2\phi_{10}$	Sphere	Sphere	−d_7_ − d_11_ − d_12_ − d_13_ − d_14_

**Table 4:** Ray incident points and ray unit vector parameters of ray path II.

I	I = 1	I = 2	I = 3	I = 4	I = 5	I = 6	I = 15	I = 16
Type	Convex Spherical	Concave Spherical	Flat Mirror (Sample)	Convex Spherical	Concave Spherical	Flat Refracting	Flat Refracting	CCD_2_
N_i_	1/1.515	1.515	Reflected	1/1.515	1.515	1/1.515	1.515	1.515
$\phi_{i}$	0	0	0	0	0	0	0	0
*n_ix_*	Cβ_1_Cα_1_	Cβ_2_Cα_2_	−C*ω_x_*S*ω_z_*	Cβ_4_Cα_4_	Cβ_5_Cα_5_	0	0	0
*n_iy_*	Cβ_1_Cα_1_	Cβ_2_Cα_2_	C*ω_x_*C*ω_z_*	Cβ_4_Cα_4_	Cβ_5_Cα_5_	1	1	1
*n_iz_*	Sβ_1_	Sβ_2_	S*ω_x_*	Sβ_4_	Sβ_5_	0	0	0
*ρ_i_*	−(d_1_ + R)	R − (d_1_ + d_2_)	0	R − (d_1_ + d_2_)	−(d_1_ + R)	0	0	0
*τ_i_*	0	0	0	0	0	0	0	0
*e_i_*	Sphere	Sphere	− (d_1_ + d_2_ + d_3_)	Sphere	Sphere	0	−d_15_	−d_15_ − d_16_
